# Bird–tick and human–tick encounters in the Rio Grande Valley (Texas, USA): ecological associations and pathogen detections

**DOI:** 10.1186/s13071-025-06725-y

**Published:** 2025-03-07

**Authors:** Julia Gonzalez, Mark Conway, Sarah A. Hamer

**Affiliations:** 1 Department of Veterinary Integrative Biosciences, Texas A&M University, College Station, TX USA; 2 Schubot Center for Avian Health, Department of Veterinary Pathobiology, Texas A&M University, College Station, TX USA; 3 Master Bird Bander, Lower Rio Grande Valley, TX USA

**Keywords:** *Amblyomma tenellum*, *Amblyomma inornatum*, *Ixodes keiransi*, Ehrlichiosis, *Rickettsia amblyommatis*

## Abstract

**Background:**

The tropical climate and diverse vector community allows the Rio Grande Valley (RGV) of South Texas to support many vector-borne pathogen transmission cycles. It is a key area for monitoring bird ticks, since most of the migratory birds fly through this corridor to move for south tropical latitudes. Some of the tick species that infest birds in Texas can also transmit tick-borne pathogens that concern public health.

**Methods:**

During bird banding activities in 2019–2024, ticks were collected opportunistically from local and migrant birds, as well as from outdoor recreationalists, to explore the presence of tick-borne pathogens. Applying a polymerase chain reaction (PCR)-DNA sequencing approach, ticks were tested for *Ehrlichia* and *Rickettsia* species.

**Results:**

Of 375 ticks, eight tick species were identified, including species regarded as locally established (*Amblyomma inornatum*, *Amblyomma maculatum*, *Amblyomma mixtum*, *Amblyomma tenellum*, and *Dermacentor variabilis*), neotropical species imported by migratory birds (*Amblyomma geayi* and *Amblyomma longirostre*), and for the first time in Texas, *Ixodes keiransi*, formerly the North American lineage of *Ixodes affinis*. *Amblyomma tenellum* was the most abundant tick species (89.3%). All ticks were screened for *Ehrlichia*, resulting in *Ehrlichia chaffeensis* detection in three *A. tenellum* ticks (one nymph and two adults) found on humans, and one positive for *Ehrlichia ewingii* in an *A. inornatum* nymph collected from a Clay-colored Thrush (*Turdus grayi*). Both bacteria can cause human ehrlichiosis, which is infrequently reported in Texas. The *Rickettsia* screening of ticks resulted in detection of *Rickettsia amblyommatis*, a potentially pathogenic spotted fever group *Rickettsia*, in nine ticks: eight *A. inornatum* ticks (one larva, five nymphs and two adults), seven of which were collected from Long-billed Thrashers (*Toxostoma longirostre*); and an *A. longirostre* engorged nymph from an Acadian Flycatcher (*Empidonax virescens*).

**Conclusions:**

Our results highlight the importance of occupational exposure to ticks and the potential public health impact of the relatively neglected human-biting vector, *A. tenellum.*. There is also a critical need to investigate the fate of bird-imported *A. inornatum* and *A. longirostre*, and the pathogens they carry.

**Graphical Abstract:**

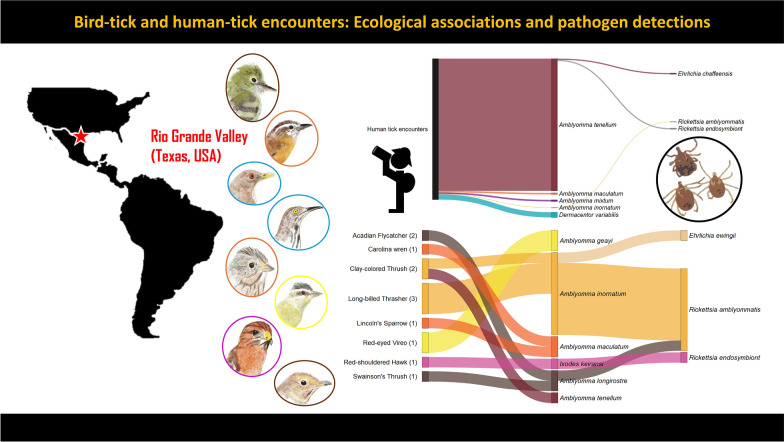

## Background

The number of tick bite cases reported in the south-central area is generally lower compared with other United States (US) regions [1]. However, its strategic Nearctic–Neotropical location as an animal migration passage, and the expansion of tick distribution caused by climate change, spotlight Texas in a focus of zoonotic diseases [2, 3]. The transboundary region of Rio Grande, a natural border between South Texas and northeast Mexico, is an area vulnerable to outbreaks owing to overlapping distributions of vectors and reservoirs of tick-borne diseases (TBDs) [2].

Given the diverse bird community along the border, and the importation of neotropical ticks on spring migratory birds arriving to the southern US [4, 5], Texas is a key area for monitoring bird ticks and their implications for human health. Most of the migratory birds fly through South Texas [6], highlighting the importance of this region for not only birds, but the parasites they harbor. Some of the tick species that infest birds in Texas, such as *Amblyomma americanum*, *Amblyomma maculatum*, and* Amblyomma inornatum* [4, 7, 8], can also parasitize humans and transmit tick-borne pathogens that concern public health. For example, the role of *A. inornatum* as a potential vector of *Ehrlichia*, *Borrelia*, and *Rickettsia* species was reported in a surveillance study of questing ticks in South Texas [7].

Several TBDs are underreported, in part because they have similar symptoms, such as fever, fatigue, muscle or joint aches, making it difficult to seek medical care and diagnoses, especially in vulnerable populations at risk for TBDs owing to occupational environments [9, 10]. Ehrlichiosis is a rare disease in Texas [11] yet the number of cases has increased in recent years [12]. The bacteria *Ehrlichia chaffeensis*, *Ehrlichia ewingii*, and *Ehrlichia muris eauclairensis* are the causative agents of ehrlichiosis in the USA, and their primary vectors are *A. americanum* and *Ixodes scapularis* ticks [13], but other tick species could be potential vectors. For instance, it was recently suggested that *Amblyomma tenellum* (formerly *Amblyomma imitator*) is a potential vector of *E. chaffeensis*, the causative agent of human monocytotropic ehrlichiosis [14].

Spotted fever group rickettsiosis (SFGR) are also reported in Texas [15], caused by bacteria including *Rickettsia rickettsii*, the agent of Rocky Mountain spotted fever, and several other species of SFGR occur in the tick populations of Texas [16]. Since new *Rickettsia* species are continuously being discovered it has suggested that we could expect the emergence of tick-borne rickettsioses [17]. Hence, it is critical to research tick populations to monitor emerging and neglected tick-borne diseases. In this study, we describe a diverse tick community and report the tick-borne microorganisms circulating through bird–tick and human–tick encounters in the Rio Grande Valley of South Texas to describe novel ecological relationships and evaluate public health risks.

## Methods

### Tick sampling and bird banding

The study was carried out in the Arroyo Colorado unit of Las Palomas Wildlife Management area (ARUN) located at 26.317° N, 97.524° W in Cameron County, an area of the Rio Grande Valley in South Texas. We collected ticks opportunistically that were attached to birds, as well as those crawling or attached to humans, during bird banding activities between 2019 and 2024. Birds were captured weekly with 4–12 mist nets (36 mm mesh) opened between sunrise and 1100 CST. Each bird was banded with a unique US Geological Survey (USGS) leg band and examined for the presence of ticks. All ticks were collected with fine-tipped forceps and placed into a microcentrifuge tube with 70% ethanol for identification.

### Tick identification

Ticks were identified according to different morphological keys [18–26] under a stereomicroscope. For confirmatory purposes, those specimens whose morphological identification was inconclusive were molecularly identified applying a PCR-DNA sequencing approach.

The DNA of individual ticks was extracted using the E.Z.N.A. tissue DNA kit (Omega Bio-Tek, Norcross, GA). Each tick was sliced with a sterile scalpel blade in a tube to open the idiosome and facilitate contact with lysis buffer. The ticks were incubated at 55 °C before completing the extraction according to the manufacturer’s instructions. For the molecular identification we performed a PCR to amplify the 12S mitochondrial ribosomal DNA (rDNA) using the T1B and T2A primers [27] and sequenced the 360-bp products (Table 1). Reactions of 15 µl were performed using 1.5 µl of extracted tick DNA with 0.75 µM of each primer, and FailSafe PreMix E buffer and enzyme (Epicentre Technologies Corp., Chicago, IL). To confirm some tick species, it was necessary to carry out additional PCRs on a subset of samples to amplify a region of the internal transcribed spacer 2 (ITS2), the cytochrome *c* oxidase subunit 1 (*COX1*) mitochondrial gene, and 16S rRNA mitochondrial gene (Table 1).

**Table 1:** Primers used for the molecular identification of tick species

Target	Primers	Sequence 5′–3′	Amplicon size	Reference
12S rDNA	T1B	AAACTAGGATTAGATACCCT	360 bp	[27]
	T2A	AATGAGAGCGACGGGCGATGT		
ITS2	ITS2-7923-F	CGGATCCTTC (A/G) CTCGCCG (C/T) TACT	1.2 kb	[70]
	ITS2-7923-R	CCATCGATGTGAA (C/T) TGCAGGAC		
*COX1*	HCO2198	GGTCAACAAATCATAAAGATATTGG	601 bp	[71]
	LCO1490	TAAACTTCAGGGTGACCAAAAAATCA		
16S rDNA	16S+1	CTGCTCAATGATTTTTTAAATTGCTGTGG	460 bp	[72]
	16S−1	CCGGTCTGAACTCAGATCAAGT		

The PCR products were visualized on 1.5% agarose gels. The positive samples were purified with ExoSAP-IT (Affymetrix, Santa Clara, CA), and Sanger sequencing was performed (Eton Biosciences, San Diego, CA). To facilitate identification, sequences were aligned and compared with a national database (NCBI Blast) using MEGA X software (2018). We considered that sequences with over 97% similarity in GenBank as belonging to the same species. Sequences of the ticks were deposited to GenBank (accession nos. PQ687577-PQ687589 for 12S, PQ699150-PQ699152 for ITS2, PQ687562 for 16S, and PQ691262 for *COX1*).

### Pathogen detection

We screened ticks for the genus *Ehrlichia* using primers Ehr DSB 330F and Ehr DSB 728R to amplify a product of 398 bp of the disulfide oxidoreductase gene *dsb* [8]. Gel electrophoresis, Sanger sequencing, and sequence comparisons were performed as previously described. Those sequences identified as *E. chaffensis* and *E. ewingii* were tested additionally with specific primers. To determine the *E. chaffeensis* genotype we targeted the variable length PCR target gene (*VLPT*) of the encoded protein TRP32 [14], and for *E. ewingii*–specific amplicons we used primers for EE52 and HE3 of the 16S ribosomal RNA gene [28], followed by DNA sequencing.

*Rickettsia* species were detected by a quantitative real-time PCR assay that amplified a partial region of the *17KD* gene using the primers R17K128 and R17K328, and the probe R17K202-FAM [29]. Confirmatory testing on the positive samples was accomplished by the amplification of a partial region of the citrate synthase gene *gltA* using the primers RrCS 372 and RrCS 989, resulting in a 617-bp product [8], followed by DNA sequencing. In addition, we targeted the outer membrane protein gene *ompA* of *Rickettsia*, commonly used to detect species of SFGR, following the semi-nested protocol described previously [30], with the primers RR190.70F and RR190.701R for the initial PCR, and RR190.602R for the second PCR.

We considered that sequences with over 98% similarity in GenBank as belonging to the same species, and for the classification of *Rickettsia* species, we followed the gene sequence-based criteria described previously [31]. Sequences of the pathogens were deposited to GenBank (accession nos. PQ730737-39 for *VLPT*, PQ730740-43 for *dsb*, PQ691265 for 16S, PQ730744-53 for *gltA*, and PQ730754-63 for *ompA*).

### Data analysis

We evaluated the tick numbers collected and calculated the tick intensity for each bird species by dividing the number of ticks collected between the number of birds infested [32] for the graphical representation. Human–tick encounters refer to the ticks found crawling or attached to humans, this concept has been previously used to measure tick-borne disease risk [33, 34]. Data visualization included in this manuscript was performed using the software R Core Team (2021).

## Results

### Tick collections

**Fig. 1:**
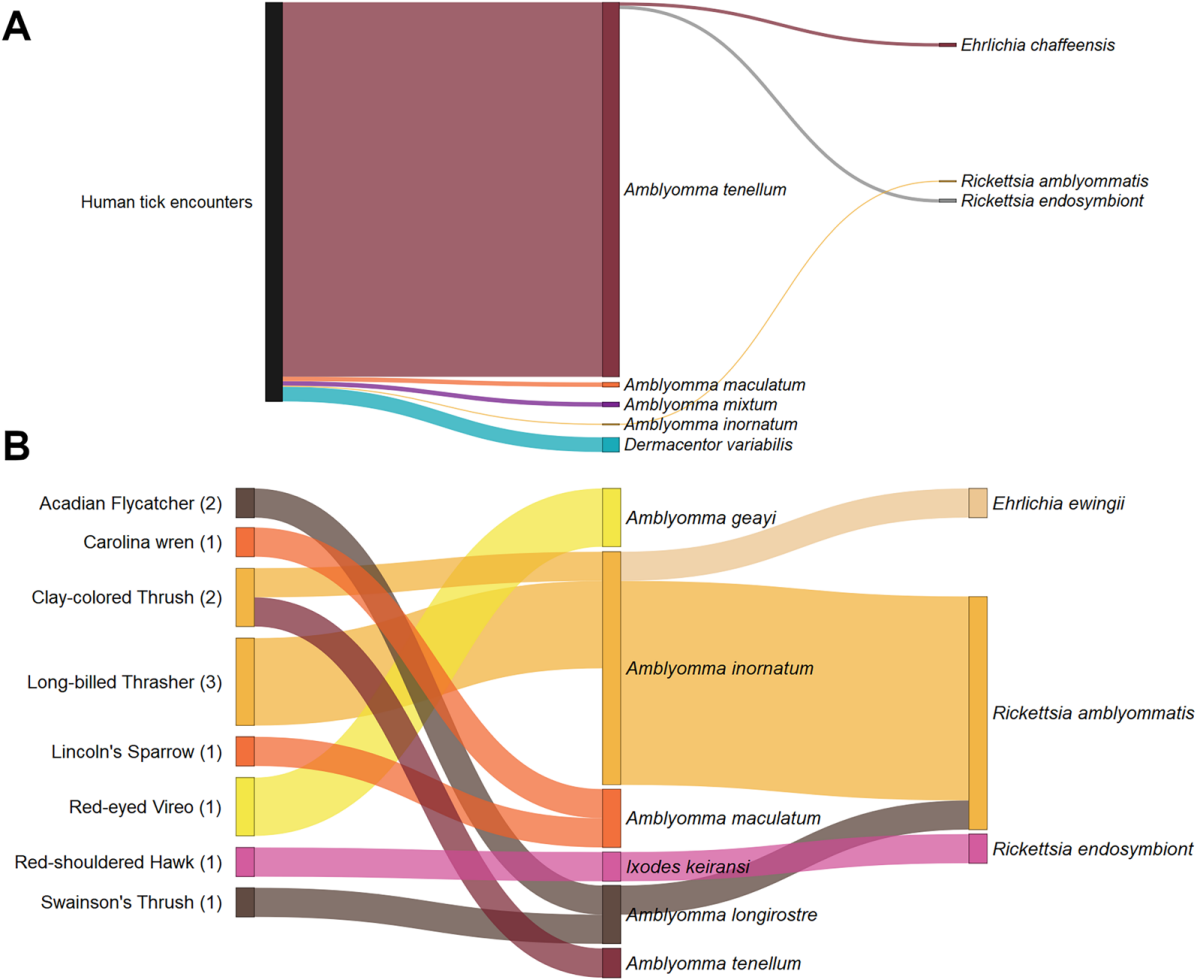
Sankey diagrams linking host–tick-pathogens detected during bird banding activities in South Texas, 2019–2024. The thickness of the lines represents the number of ticks. **A** Ticks found crawling or attached to humans (*n* = 356 ticks). **B** Tick intensity on different bird species examined (*n* = 19 ticks); sample size of birds is shown between parentheses

We morphologically identified 375 ticks (39 larvae, 151 nymphs, and 185 adults) belonging to eight tick species (Fig. 1); for confirmatory purposes a subset of 82 ticks were also molecularly identified. *Amblyomma tenellum* was the most abundant species (89.3%) in the collection, followed by *Dermacentor variabilis* (3.5%), *A. inornatum* (2.9%), *A. maculatum* (1.6%), *Amblyomma longirostre* (0.8%), *Amblyomma geayi* (0.5%), *Amblyomma mixtum* (1.1%; included in the *Amblyomma cajennense* complex), and *Ixodes keiransi* (0.3%).

The majority of ticks collected were found in human–tick encounters (94.9%), including 347 ticks found crawling and 9 *A. tenellum* specimens that were attached to the bird-banders (Table 2). In parallel, 19 ticks were collected from 12 birds of 8 different species (Table 3): Acadian Flycatcher (*Empidonax virescens*), Carolina Wren (*Thryothorus ludovicianus*), Clay-colored Thrush (*Turdus grayi*), Lincoln’s Sparrow (*Melospiza lincolnii*), Long-billed Thrasher (*Toxostoma longirostre*), Red-eyed Vireo (*Vireo olivaceus*), Red-shouldered Hawk (*Buteo lineatus*), and Swainson’s Thrush (*Catharus ustulatus*).

**Table 2:** Ticks found crawling or attached to humans during banding activities between 2019 and 2024, Rio Grande Valley of South Texas

Tick species	Tick collected	January	February	March	April	May	June	July	August	September	October
*Amblyomma inornatum*	Crawling								1 (N)		
*Amblyomma maculatum*	Crawling			1 (N)						2 (A)	1 (A)
*Amblyomma mixtum*	Crawling								1 (A)	3 (A)	
*Amblyomma tenellum*	Attached				5 (4N, 1A)	2 (A)	1(N)	1 (A)			
	Crawling			51 (33L, 15N, 3A)	86 (65N, 21A)	71 (37N, 34A)	46 (6N, 40A)	36 (10N, 26A)	25 (1N, 24A)	10 (A)	
*Dermacentor variabilis*	Crawling	1 (A)		2 (A)			2 (A)		4 (A)	1 (A)	3 (A)
**Total**		**1**		**54**	**91**	**73**	**49**	**37**	**31**	**16**	**4**

Total numbers are highlighted in bold

Numbers represent the sum of ticks found each month during the study years. Blank spaces mean no ticks were collected. *L* larva, *N* nymph, *A* adult

**Table 3:** Ticks collected from birds during banding activities between 2019 and 2024, Rio Grande Valley of South Texas

Birds (*n*)	Migratory status	Capture date	*Amblyomma geayi*	*Amblyomma inornatum*	*Amblyomma longirostre*	*Amblyomma maculatum*	*Amblyomma tenellum*	*Ixodes keiransi*
ACFL (2)	B, LD	25 April 2023			2 (L, N)			
CARW (1)	B, NM	25 February 2024				1 (N)		
CCTH (2)	B, NM	1 October 2023, 21 April 2024		1 (N)			1 (N)	
LBTH (3)	B, NM	3 September, 18 August 2024		9 (2L, 4N, 3A)				
LISP (1)	NB, MD	4 January 2023				1 (N)		
REVI (1)	B, LD	12 May 2019	2 (L)					
RSHA (1)	B, NM	1 February 2020						1 (N)
SWTH (1)	NB, LD	19 May 2019			1 (L)			
		**Total**	**2**	**10**	**3**	**2**	**1**	**1**

Total numbers are highlighted in bold

*ACFL* Acadian Flycatcher, *CARW* Carolina Wren, *CCTH* Clay-colored Thrush, *LISP* Lincoln’s Sparrow, *LBTH* Long-billed Thrasher, *REVI* Red-eyed Vireo, *RSHA* Red-shouldered Hawk, *SWTH* Swainson’s Thrush *B* breeding in Texas, *NB* non-breeder in Texas, *LD* long-distance migrant, *MD* medium-distance migrant, *NM* non-migratory, *L* larva, *N* nymph, *A* adult. References used to include the migratory status are listed [6, 54, 73–76]. Blank spaces mean none infested

Nine *A. inornatum* ticks, including three adults, were collected in three Long-billed Thrashers, a bird species that lives year-round in southern Texas. Furthermore, we found two nymphs, identified as *A. inornatum* and *A. tenellum*, on two Clay-colored Thrushes, and an *A. maculatum* nymph on a Carolina Wren, both bird species are also year-round residents. In winter, we collected an *A. maculatum* nymph from a Lincoln’s Sparrow, a medium-distance migrant. Curiously, one *I. keiransi* nymph was found on a Red-shouldered Hawk also during this season. Moreover, three long-distance migratory species from South America—the Acadian Flycatcher, Red-eyed Vireo, and Swainson’s Thrush—were trapped during spring migration, and infested with *A. longirostre* and *A. geayi* ticks.

### Pathogen detection

**Fig. 2:**
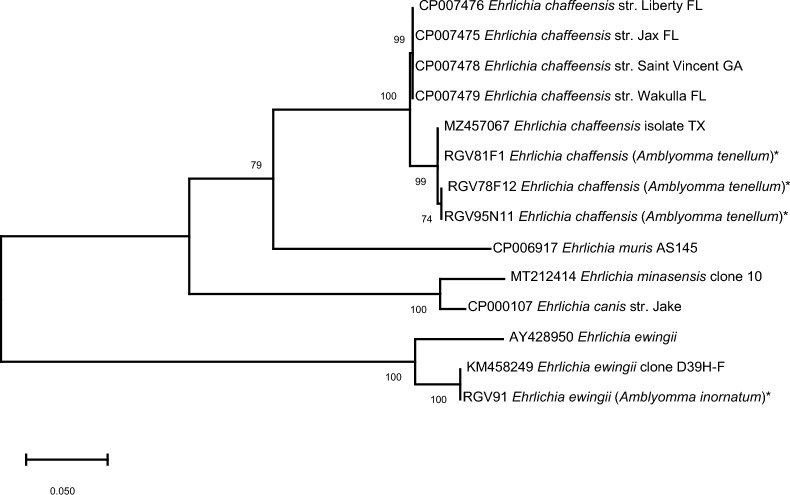
Phylogenetic tree of the *dsb* gene from *Ehrlichia* using the maximum likelihood method and Tamura–Nei model [41]; log likelihood − 1366.46. Sequences obtained in this study are marked with an asterisk (*). The percentage of trees in which the associated taxa clustered together is shown next to the branches. Initial tree(s) for the heuristic search were obtained automatically by applying Neighbor-Join and BioNJ algorithms to a matrix of pairwise distances estimated using the maximum composite likelihood (MCL) approach, and then selecting the topology with the superior log likelihood value. The rate variation model allowed for some sites to be evolutionarily invariable ([+ I], 33.54% sites). The tree is drawn to scale, with branch lengths measured in the number of substitutions per site. There was a total of 404 positions in the final dataset. Evolutionary analyses were conducted in MEGA X [42]

We found that 1.1% (4/375) of the ticks were positive for an *Ehrlichia* species (Clopper–Pearson exact 95% CI 0.3–2.7%). Three *A. tenellum* ticks (0.9%), two unfed females and one nymph, found crawling on humans contained *dsb* gene sequences that were 99–100% identical to *E. chaffeensis* detected in *A. tenellum* ticks from South Texas (GenBank accession MZ457067 [14]. A phylogenetic tree of this gene comparing *E. chaffeensis* sequences available in GenBank with the positive samples obtained in this study showed our results are closely related to the sequence reported in South Texas (Fig. 2). Additional sequencing of the *VLPT* gene, encoding the protein TRP32, confirmed the same species of *E. chaffeensis* in those samples (GenBank accession MZ457069) [14] with 98% identity.

Moreover, a nymph of *A. inornatum* collected from a Clay-colored Thrush contained DNA 99% similar to the *dsb* region from *E. ewingii* (GenBank accession KM458249; [7]) (Fig. 2), and 100% 16S rRNA identity to the same species through a confirmatory analysis (MN336353; [35]).

**Fig. 3:**
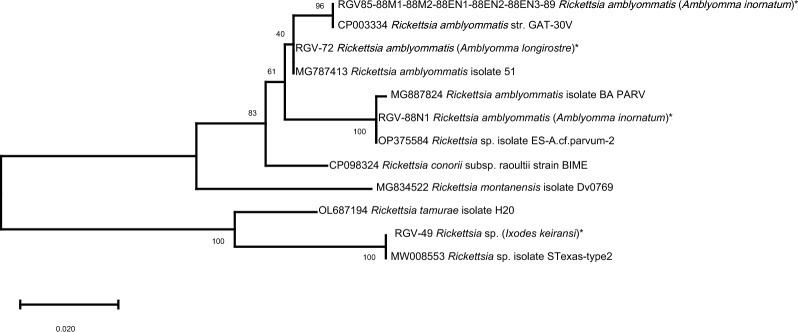
Phylogenetic tree of the *ompA* gene of *Rickettsia* species using the maximum likelihood method and the general time reversible model [51]; log likelihood -1213.84. Sequences obtained in this study are marked with an asterisk (*). The percentage of trees in which the associated taxa clustered together is shown next to the branches. Initial tree(s) for the heuristic search were obtained automatically by applying Neighbor-Join and BioNJ algorithms to a matrix of pairwise distances estimated using the maximum composite likelihood (MCL) approach, and then selecting the topology with the superior log likelihood value. A discrete gamma distribution was used to model evolutionary rate differences among sites [two categories (+ G, parameter = 0.3509)]. The tree is drawn to scale, with branch lengths measured in the number of substitutions per site. Codon positions included were 1st + 2nd + 3rd + noncoding. There was a total of 502 positions in the final dataset. Evolutionary analyses were conducted in MEGA X [42]

Overall, 3.5% (13/375) of the ticks were positive for a *Rickettsia* species (Clopper–Pearson exact 95% CI 1.8–5.8%). Eight out of eleven (72.7%) *A. inornatum* ticks produced *gltA* and *ompA* sequences with 99–100% identity to *Rickettsia amblyommatis* (GenBank accession CP003334; [36]) or a closely related *Rickettsia* species (*gltA* sequence GenBack accession MK112516 [37]; and *ompA* sequence GenBank accession OP375584 [38]) (Fig. 3). All these *A. inornatum* were collected from two Long-billed Thrasher birds, except one engorged nymph that was found crawling. We also detected *R. amblyommatis* in an engorged nymph of *A. longirostre* collected from an Acadian Flycatcher with a 100% match (*ompA* sequence MG787413; [39]. Four ticks were positive for other *Rickettsia* species. Three *A. tenellum* ticks, found crawling, that contained *gltA* sequences 91% similar to the *Rickettsia* endosymbiont *Ixodes boliviensis* (MW699694; [40]), and a nymph identified as *I. keiransi* collected from a Red-shouldered Hawk that contained a sequence 98% similar to the *ompA* sequence from the *Rickettsia* sp. isolate STexas-type2 (rickettsial endosymbiont from *I. scapularis;* MW008553 [41]) (Fig. 3) and 99.5% identity to the *gltA* sequence from an uncultured *Rickettsia* species (MT441702 [42]).

## Discussion

*Amblyomma tenellum* was the most abundant tick found on humans in this study in the RGV of South Texas; this tick species was redescribed as a valid species from southern Texas and Mexico, formerly named *A. imitator* [24] when it was considered as synonym of *A. cajennense* [25]. The most recent taxonomic studies [43, 44] redefined and updated the species related to the complex taxon *A. cajennense*, including *A. cajennense* sensu stricto, *Amblyomma sculptum*, *A. mixtum*, *Amblyomma tonelliae*, *Amblyomma interandinum*, and *Amblyomma patinoi*. Thus, the reexamination of material resulted in exclusion of *A. tenellum* from this group despite its similarity. In fact, *A. tenellum* nymphs are often confused with *A. mixtum* and *A. americanum*, as their distributions overlap in Texas [18], but it is genetically more closely related to *A. americanum* than to the *A. cajennense* group [43]. Apparently, *A. tenellum* specimens are likely to be found in southern Texas, i.e., Cameron and Hidalgo counties, feeding on a great diversity of hosts, including birds (Passeriformes: Mimidae and Turdidae) and humans, albeit sporadically [45]. Indeed, it was the only species found questing in a recent study conducted on the southern tip of coastal Texas [14], showing its predominance in the region. Most *A. tenellum* collected in our study were found crawling, likely encountered by humans while setting up or taking down mist netting equipment or checking nets for birds, as these activities place people close to vegetation where ticks may be questing. This scenario underscores the importance of checking for ticks in time, since they can be removed before attachment.

*Amblyomma tenellum* is a species of public health concern that has been reported as a potential vector of *R. rickettsii*, the agent of Rocky Mountain spotted fever [46], and recently a novel *E. chaffeensis* genotype (a cause of human monocytotropic ehrlichiosis) has been characterized from this tick species [14]. The *E. chaffeensis* sequences obtained in this study were more closely related to this novel genotype than other strains reported in the National Center for Biotechnology Information (NCBI), which may be expected given the studies were conducted in the same region. The *Ehrlichia* infection prevalence we report (0.9%) is comparable with other studies carried out in Texas (detections of *E. chaffeensis* in *A. americanum* ticks are reported from 0.2% to 8.8% [16, 35]), but it was higher than that previously reported for *A. tenellum* (0.1%) [14]. Our study, and that of [14], confirm the sustained presence of *E. chaffeensis* in southern Texas despite the lack of reported human ehrlichiosis cases. The occurrence of *E. chaffeensis*-infected unfed *A. tenellum* ticks (2 adults and 1 nymph) suggests that the bloodmeal hosts of the prior life stages served as the sources of infection to the ticks, given this bacterium is not known to be transovarially transmitted. White-tailed deer—which are abundant in South Texas—are a natural reservoir of *E. chaffeensis* [47], yet the role of deer or other animals in the enzootic maintenance of *E. chaffeensis* in South Texas has not been studied.

Interestingly, an increasing trend of canine ehrlichiosis has been documented in Texas [9, 48], apparently driven more by the seroprevalence of *Ehrlichia canis* transmitted by *Rhipicephalus sanguineus*, than *E. chaffeensis* and *E. ewingii,* which are transmitted mainly by *A. americanum* [49, 50], but are also detected in *A. mixtum*, *A. maculatum*, and *A. inornatum* [7, 8]. Cross-reactivity between *Ehrlichia* species on commercial diagnostic tests could explain this scenario of overlapping ehrlichiosis cases without knowledge of which tick vectors are causing these changes [50].

*Amblyomma inornatum* is a common species in southern Texas and Mexico [21, 22], where it finds a wide range of hosts to feed on, including birds [21]. It was the most prevalent tick species collected from birds in this study, especially non-migratory birds that breed in Texas. They also feed on humans, which led to a study of their role as a potential vector of pathogens of public health interest in Texas, finding *Ehrlichia* species in 7.1% of *A. inornatum* ticks [7]. We detected *E. ewingii* in an *A. inornatum* nymph collected from a Clay-colored Thrush, showing that *Amblyomma* species other than *A. americanum* may be involved in the maintenance of this pathogen of concern for public health and animal health. Indeed, it has been suggested that since the seasonal activities of *A. inornatum* and other *Amblyomma* species overlap in South Texas, co-feeding could explain the rate of pathogen infection in this tick species [7]. In addition, a high seroprevalence of canine ehrlichiosis has been reported in South Texas, likely owing to the inclusion of *E. ewingii* in the diagnostic tests [51]. Hence, further studies would be useful to know which tick species and *Ehrlichia* species are circulating in this area to understand the risk of tick infestation.

Prior studies of ticks on birds in Texas include work during spring migration at a high passage coastal stopover site along the Gulf of Mexico; this work showed 3.6% of 3844 birds harbored ticks, including seven different *Amblyomma* species and a single *Ixodes* species [4]. In East Texas, only 1.9% of 211 birds harbored ticks, exclusively *Ixodes dentatus* and *I. scapularis* [52]. More recent work in the northern Gulf of Mexico reported a lower tick prevalence (< 1%) in 17,550 birds sampled in Louisiana and Alabama, with *Amblyomma* being the most abundant tick genera and *A. longirostre* the most abundant species [5]. A much higher tick infestation prevalence of birds was detected one state north in Oklahoma, with 24.2% of 459 birds harboring ticks of three species; *A. americanum*, *A. maculatum*, and *Haemaphysalis leporispalustris* [53]. In the current study, the tick infestation prevalence in birds was not determined, but the richness of species present (six tick species) among the relatively small sample size of ticks removed from birds (19 ticks) suggests a high tick biodiversity.

*Rickettsia amblyommatis* was detected in 88.9% of *A. inornatum* ticks, collected mostly from two Long-billed Thrashers, a non-migratory passerine of dry and brushy landscapes of southeast Texas and northeastern Mexico [54]. Our data, combined with others, confirms this tick and pathogen are established in Texas [4, 7, 16, 35, 41, 55]. *Rickettsia amblyommatis* is a bacterium belonging to the spotted fever group, widely recorded in several tick species from the Neotropical region [56], and mainly detected in *A. americanum* across the USA [36]. Besides *A. inornatum*, we also found *R. amblyommatis* in one *A. longirostre* nymph collected from an Acadian Flycatcher, a long-distance migratory species. It is therefore not surprising that the role of migratory birds carrying ticks, such as *A. longirostre*, has been suggested as one of the main routes of dispersal of this bacterium [56, 57]. Studies from South and Central America have also reported this tick–bird association and the detection of *R. amblyommatis*, discussing the role they might play on the expansion of rickettsial pathogens [39, 58]. As recently reviewed [59], the presence of *R. amblyommatis* may prevent ticks from acquiring other *Rickettsia* species [60], an interaction that has caused changes in the epidemiology of SFGR in the USA [61]. Several studies have also shown that when *R. rickettsii* is co-infected with *R. amblyommatis*, the latter can lower the severity of Rocky Mountain spotted fever (RMSF) symptoms [59]. However, *R. amblyommatis* single-infection can provoke a vascular inflammation and mild febrile illness in mammals [62–65] with RMSF-like symptoms, although its potential pathogenicity in humans is still unknown.

The detection of putative *Rickettsia* endosymbionts in four ticks warrants further investigation, as nonpathogenic microorganisms may impact their tick host or alter the transmission of tick-borne pathogens [66]. The interactions of pathogenic species with rickettsial endosymbionts may help explain why rickettsioses are still emerging worldwide [17]. Interestingly, one of the *Rickettsia* endosymbionts was found in an *I. keiransi* nymph collected from a Red-shouldered Hawk. This tick species was previously described as a North American population of *Ixodes affinis*, but it has been recently considered as a distinct species [67]. Its potential distribution includes the southeastern USA, nonetheless this study is the first official record of *I. keiransi* in Texas. The specimen was morphologically similar to the description of *I. affinis* nymphs [68]; however, some characteristics were not clearly observed; i.e., the auriculae were not pronounced and the triangular internal spur was not broadly distinguished. Therefore, it was analyzed molecularly to confirm tick identity following the protocol recently described [69]. According to the latest studies [67], *I. affinis* s.s. is not found in the USA, thus those descriptions attributed to *I. affinis* could indeed correspond to *I. keiransi*, although further comparative studies are needed to describe formally the morphology of its immature stages. Given the Red-shouldered Hawk on which this *Rickettsial* endosymbiont-infected tick was found is likely to be a non-migratory permanent resident of South Texas, this tick and its bacterial community are probably locally established.

## Conclusions

Our findings provide key information on tick and pathogen species that interact with humans and native avian wildlife, and highlights the human risk of tick and pathogen encounters during occupational outdoor activities, such as bird banding. Migratory birds are one of the main routes of tick dispersal, transporting tick species and pathogens to new locations. Our study also underscores the importance of clarifying the roles of the relatively neglected human-biting *A. tenellum* and bird-imported ticks *A. inornatum* and *A. longirostre* in the transmission of emerging and neglected tick-borne diseases to establish the necessary prevention measures to avoid tick bites.

## Data Availability

The data supporting this study is provided within the article, including the accession numbers of representative sequences submitted in the GenBank database.
